# Analgesic therapy failure in a *COMT *HPS/HPS diplotype carrier heterozygous for the *CYP2D6* *4 allele with fibromyalgia—a case report

**DOI:** 10.1097/PR9.0000000000001248

**Published:** 2025-02-21

**Authors:** Anna Bollinger, Julia Gianora, Tanja Schüpbach, Samuel S. Allemann, Céline K. Stäuble, Henriette E. Meyer zu Schwabedissen

**Affiliations:** a Biopharmacy, Department of Pharmaceutical Sciences, University of Basel, Basel, Switzerland; b Pharmaceutical Care Research Group, Department of Pharmaceutical Sciences, University of Basel, Basel, Switzerland; c Institute of Hospital Pharmacy, Solothurner Spitäler AG, Olten, Switzerland

**Keywords:** Chronic pain patient, Pharmacogenetics, PGx panel test, High pain sensitivity, Clinical practice

## Abstract

**Introduction:**

The cytochrome P450 enzyme 2D6 (CYP2D6) and the catechol-O-methyltransferase (COMT) enzyme are involved in catecholamine metabolism, potentially influencing pain modulation. Catechol-O-methyltransferase has 3 major haplotypes related to pain sensitivity: low (LPS), average (APS), and high (HPS). However, the reliability of these haplotypes in predicting clinical outcomes is not well investigated. We present a 40-year-old female patient with fibromyalgia. Despite extensive pharmacotherapy with 120 mg/d duloxetine, 150 mg/d pregabalin, 80 mg/d oxycodone, 2 g/d paracetamol, and 1.6 g/d ibuprofen, she suffered from severe pain.

**Objectives:**

We aim to investigate the patient’s susceptibility to analgesic therapy failure (TF) and pain sensitivity with pharmacogenotyping.

**Methods:**

PGx panel testing, including CYP2D6 and COMT rs4680, was conducted by a commercial provider. Additional genotyping of COMT rs6269, rs4633 and rs4818 was performed applying PCR, restriction fragment length polymorphism assay and sanger sequencing.

**Results:**

The patient was identified as COMT HPS/HPS diplotype carrier and CYP2D6 intermediate metabolizer. CYP2D6 is mainly responsible for the bioactivation of oxycodone into oxymorphone. Reduced CYP2D6 activity may result in a lower oxycodone activation. Considering the coadministration of duloxetine (a moderate CYP2D6 inhibitor), the TF of oxycodone could also be the result of a drug–drug–gene interaction. No other medications were affected by her genetic profile.

**Conclusion:**

We hypothesize that the broad TF of pain medications and associated high pain sensitivity could be related to the patient’s genetic predisposition in CYP2D6 and COMT, warranting further investigation in a larger patient sample.

## Abstract 2

Supplemental Digital Content is Available in the Text.

Genetic predispositions in *CYP2D6* and catechol-O-methyltransferase contribute to high pain sensitivity and analgesic therapy failure in a fibromyalgia patient, suggesting the need for further study.

## 1. Introduction

Pain is the most characteristic symptom of numerous conditions and diseases. The selection of an analgesic drug as well as the dosage are usually standardized. However, response to pharmacotherapy is not equal in every patient.^16^

One endogenous factor contributing to interindividual drug responses is variation in genes that are involved in the pharmacokinetics (PK) and pharmacodynamics (PD) of a drug.^16^ Such genetic variants can be detected with a pharmacogenetic (PGx) test. Pharmacogenetic panel testing can be used to identify the inherited component of an atypical drug response and allows to provide recommendations for PGx actionable drugs.^17^ This approach can be particularly useful in pain medicine since the metabolism of various analgesic drugs is influenced by genes that exhibit a PGx variability. For example, variations in *CYP2D6* were identified to alter response to codeine, tramadol, and oxycodone. Since these prodrugs are bioactivated involving CYP2D6, individuals with 1 normal allele and 1 nonfunctional allele or 2 nonfunctional alleles can result in an intermediate metabolizer (IM) or poor metabolizer (PM) phenotype. Those phenotypes would clinically manifest in a decreased activation of the parent substances to morphine, O-desmethyltramadol, or oxymorphone, respectively, with the expected result of less pain relief.^3,21^ In the context of neuropathic pain, it seems noteworthy that the response to antidepressants used as pain-modulating coanalgesics can also depend on genetic variations, especially in *CYP2D6* and *CYP2C19*.^7^ However, not only variations in the CYPs but also variations in receptors (eg, opioid receptor μ1 [*OPRM1*]), transporters (eg, ATP binding cassette subfamily B member 1 [*ABCB1*]), and phase-II-enzymes (eg, catechol-O-methyltransferase [*COMT*]) are associated with the interindividual response to analgesics.^3^

Besides the influence on the PK and PD of analgesics and opioids, individuals' genetic variations may also affect pain sensitivity. It is known that the polymorphic CYP2D6 is involved in the synthesis of catecholamine.^14^ Moreover, it is assumed that CYP2D6 contributes to the biosynthesis of endogenous morphines (aka endorphins).^19^ Some studies report that a lower CYP2D6 activity (IM or PM) is associated with a higher pain sensitivity for different types of pain, whereas a higher CYP2D6 activity, found in ultrarapid metabolizers (UM), is associated with a lower pain sensitivity.^14,19^

Also, the *COMT* is involved in the physiological process of pain modulation, as it regulates the concentration of catecholamine and enkephalin. Accordingly, changes in *COMT* activity result in altered levels of catecholamine and enkephalin, which may influence pain sensitivity.^12,20^ Variations in *COMT* single nucleotide polymorphisms (SNP) rs4680, rs6269, rs4633, and rs4818 can be assigned to 3 major haplotypes, which relate to an individual's responsiveness to pain: low pain sensitive (LPS), average pain sensitive (APS), and high pain sensitive (HPS).^2,5^ The reliability of these haplotypes predicting a clinical outcome for pain control has not yet been well investigated.

However, PGx testing may be used to determine the genotype-predicted phenotype of *CYP2D6* and *COMT*, which might contribute to an objective assessment of the individual's pain sensitivity.

To demonstrate the application of PGx test results in evaluating analgesic TF and providing recommendations for pharmacotherapies, we present this case.

## 2. Methods

### 2.1. Clinical case

A 40-year-old female patient was diagnosed with chronic lumbalgia and fibromyalgia. In addition, she had a moderate recurrent depressive, as well as a dissociative disorder and, therefore, multiple drug therapies. After excluding that her current medication plan exhibits potential drug–drug interactions (DDI), failure of her pain therapy was the decisive factor for initiating PGx testing. Despite highly extended pharmacotherapy (Table 1),^1^ the patient still reported insufficient pain relief, on which we focus in this report. To investigate the genetic association of the patient's susceptibility to analgesic TF, a PGx panel test was conducted.

**Table 1:** Patient's analgesic pharmacotherapy.

Substance	Dosage
Duloxetine 60 mg	2-0-0-0
Pregabalin 50 mg	1-1-0-1
Oxycodone/naloxone retard 40/20 mg	1-0-1-0
Paracetamol 500 mg	As required, max. 1-1-1-1
Ibuprofen 400 mg	As required, max. 1-1-1-1

### 2.2. Pharmacogenetic analysis

Pharmacogenetic panel testing of 96 polymorphisms in 30 different genes, including *CYP2D6* and *COMT* rs4680, was conducted by the commercial provider Stratipharm by humatrix AG (Pfungstadt, Germany). Additional genotyping of *COMT* rs6269, rs4633, and rs4818 was performed by applying polymerase chain reaction (PCR) with subsequent Sanger sequencing, or restriction fragment length polymorphism (RFLP) assay, using the patient's genomic DNA isolated from a whole blood sample (Supplementary Material, http://links.lww.com/PR9/A285).

## 3. Results

The patient was identified as *CYP2D6* IM (*4 heterozygous carrier). In addition, genotyping of the *COMT* identified the patient as HPS/HPS diplotype carrier (Table 2). Pharmacogenetic testing results of further genes potentially relevant in the PK and PD of opioids, other analgesics, and coanalgesics are summarized in the Supplementary Material, http://links.lww.com/PR9/A285.

**Table 2:** Selected pharmacogenetic results with phenotype interpretation.

Gene	Variant	Genotype	Diplotype	Interpretation
CYP2D6	rs1065852 NM_000106.4:c.100C>T (in *4,*10) rs3892097 NM_000106.4:c.506-1G>A (in *4)	C/T G/A	*1/*4	Intermediate metabolizer (IM) with reduced enzyme activity
COMT	rs6269 NG_011526.1:g.25690A>G rs4633 NG_011526.1:g.25973C>T rs4818 NG_011526.1:g.26945C>G rs4680 NM_000754.4:c.472G>A	A/A C/C C/C G/G	HPS/HPS	*COMT* HPS/HPS diplotype carrier implicating lower *COMT* activity and higher pain sensitivity

COMT, catechol-O-methyltransferase; HPS, high pain sensitive; IM, intermediate metabolizer.

## 4. Discussion

We focused on the evaluation of the patient's *CYP2D6* and *COMT* genotyping results. The patient was identified as heterozygous *CYP2D6* *4 allele carrier, predicting an IM status, which might have an impact on her current medication. Studies reported that reduced CYP2D6 enzyme activity can lead to a decreased bioactivation of oxycodone with lower formation of the active metabolite oxymorphone.^15,21^ Even if this drug–gene interaction (DGI) describes an established PK relationship between oxycodone and *CYP2D6*, the evidence about thereon-based clinical outcomes is limited.^3^ However, considering the fact that duloxetine is coadministered, the failure in pain relief could even be a consequence of a drug–drug–gene interaction (DDGI).

Duloxetine is a moderate inhibitor of CYP2D6, so it might have further suppressed the already reduced activity in CYP2D6, converting the patient from IM towards PM status, a phenomenon known as phenoconversion.^9^ As a result, there would have been even less metabolic capacity for the CYP2D6-mediated bioactivation of oxycodone, possibly contributing to the observed low pain relief despite extended analgesic therapy.

Overall, the ineffectiveness of the first-line therapy with duloxetine and pregabalin cannot be explained by the observed genetic profile according to current knowledge. The same applies to paracetamol and ibuprofen (Supplementary Table 1, http://links.lww.com/PR9/A285). Due to the patient's CYP2D6 IM status, it was recommended to switch to another opioid, whose metabolism is not influenced by CYP2D6 (eg, tapentadol, morphine), which consequently also prevents the DDGI with duloxetine. From medical records 1 year after the PGx test, it can be inferred that the patient and involved physician decided to continue with oxycodone, but instead switched duloxetine to venlafaxine, which was not our first choice recommendation.

Based on the PGx test results, both duloxetine and pregabalin are first choice coanalgesics, as the metabolism of other coanalgesics (eg, trimipramine, venlafaxine) is associated with increased risk for ADRs in CYP2D6 IM patients.^7^ However, medical records showed no additional analgesics in the patient's medication plan, indicating a sufficient therapy effect from continuing oxycodone and replacing duloxetine with venlafaxine. Venlafaxine is a weak CYP2D6 inhibitor and compared to duloxetine less likely to alter the enzyme's activity.

Besides its role in drug metabolism, CYP2D6 could also be considered in the assessment of the patient's pain sensitivity itself. A higher pain sensitivity found in CYP2D6 IMs and PMs fits the clinical presentation of the patient.^14,19^ Moreover, our patient was identified as *COMT* HPS/HPS diplotype carrier. The *COMT* HPS/HPS diplotype is expected to have lower enzymatic activity and is, therefore, assumed to be associated with higher pain sensitivity.^8,12^ This was previously reported in patients with fibromyalgia.^10,18^

Although it is well known that *COMT* plays a role in pain modulation, the evidence on whether specific variations contribute to an altered pain sensitivity remains conflicting and inconsistent. While there are reports suggesting that variability in the *COMT* SNP rs4680 c.472 G>A is accountable for different outcomes of pain sensitivity,^13,20^ there are others suggesting that the prediction of pain sensitivity is based on *COMT* haplotypes, which were shown to be associated with different sensitivities for experimental pain.^4,5^ According to Diatchenko et al.,^5^ there are 3 polymorphisms within the *COMT* gene (rs6269, rs4633, rs4814) that form a haploblock with rs4680, which underlie the LPS/APS/HPS haplotypes.

For the noncoding SNP rs6269 and the synonymous SNPs rs4633 and rs4818, it is assumed that they may have an influence on maintaining mRNA secondary structure and mRNA stability, respectively.^12^ Studies suggested that it is rather the combination of the 4 SNPs of the *COMT* haploblock than the single SNPs that have a synergistic effect on *COMT* function determining the functional outcome of pain.^2,11^ Considering the later findings on the haplotypes, we hypothesize that the patient's *COMT* HPS/HPS haplotype may be of relevance for her therapeutic outcome.

Notably, pain is of great subjective nature and is influenced by numerous factors (eg, environmental, psychological, sex), which also might have an impact on the individual's pain sensitivity, especially since the patient had a background of mental and behavioral disorders, which can trigger somatoform pain conditions.^6^

In conclusion, besides the identified DDGI potentially lowering the efficacy of oxycodone, it was not possible to explain TF by the genetic profile. Here, we assume that the observed broad TF on pain relief could be attributable to the patient's genetic predisposition in *CYP2D6* and *COMT* and the associated high pain sensitivity. This hypothesis needs to be validated by further investigations in a larger patient sample, as to our knowledge to date no study has simultaneously investigated the role of *CYP2D6* and *COMT* in pain perception.

## Disclosures

The authors report no conflicts of interest in this work and have no relevant affiliations or financial involvement with any organization.

## Appendix A. Supplemental digital content

Supplemental digital content associated with this article can be found online at http://links.lww.com/PR9/A285.
