# Supplementary material for: Analgesic therapy failure in a COMT HPS/HPS diplotype carrier heterozygous for the CYP2D6 *4 allele with fibromyalgia—a case report
Source: Pain Rep. 2025 Feb 21;10(2):e1248. doi: 10.1097/PR9.0000000000001248 (PMC11850035; doi:10.1097/PR9.0000000000001248)
Supplement: Supplementary file 1 [file painreports-10-e1248-s001.pdf]

SUPPLEMENTARY MATERIAL

Protocol: DNA Isolation and *COMT* genotyping

Genomic DNA was isolated using 250 µl of ethylene diamine tetraacetic acid (EDTA) blood samples, the QIAamp® DNA Blood Mini Kit and the QIACube® following the manufacturer's instructions (Qiagen, Hilden, Germany). The concentration of the extracted DNA was determined photometrically using the Tecan infinite® M200 Pro (Tecan, Männedorf, Switzerland). The sample had to reach the 260/280nm-ratio of absorbance of 1.8 and 2.05 to be applicable for genotyping.

COMT rs6269 and rs4633 were determined by Sanger sequencing, while restriction fragment length polymorphism (RFLP) analysis was applied for *COMT* rs4818. Prior to the sequencing and the restriction digest a 1563 bp amplicon was generated by polymerase chain reaction (PCR).

The 50 µl PCR reaction mix consisted of 1x Taq buffer, 2 mM MgCl2, 0.2 mM dNTP's, 1.25 U Taq Polymerase recombinant (Invitrogen by Thermo Fisher Scientific, Carlsbad, USA), 0.5 µM forward primer 5`-GCCAGAGGCACACACCTGCTC-'3 and reverse primer 5`-CAGTGAACGTGGTGTGAACACCTGG-'3 (Microsynth AG, Balgach, Switzerland) and 20 ng genomic DNA. The program of the T100TM Thermal Cycler (Bio-Rad Laboratories Inc., Hercules, USA) was: 94°C for 3 min, followed by 50 cycles of 30 sec at 94°C, 30 sec at 70.2°C and 1 min of 72°C and finished with final 10 min at 72°C.

Successful amplification was verified by 0.8% agarose gel electrophoresis, followed by purification of the sample applying the NucleoSpin Gel and PCR Clean-up kit (Macherey-Nagel, Düren, Germany) following the manufacturer's instructions. The DNA concentration was determined photometrically prior to submission of the sample to Microsynth AG for sequencing. The sequencing primer was 5'-GATAACAGCTTCTCCTGTAAG-`3 (Microsynth AG). 300 ng of the purified PCR amplicon was applied to a restriction digest for 45 min at 37°C with BclI enzyme using the FastDigest chemistry (Thermo Fisher Scientific, MA, USA). Fragment sizes were determined by 0.8% agarose gel electrophoresis. The restriction resulted in the two fragments of 1382 bp and 181 bp size, which were indicative for the presence of the G-Allele in rs4818. A 1kb DNA Ladder (Thermo Fisher Scientific) was used for size determination.

Supplementary Table 1. Further pharmacogenetic (PGx) testing results regarding the metabolism of analgesics, opioids, and co-analgesics.

Gene	Variant<br>also tested variants in gene locus	Genotype	Diplotype	Interpretation
ABCB1	rs1045642 c.3435T>C<br>rs1128503 c.1236T>C<br>rs2032582 c.2677G>T<br>(rs2032583)	C/T<br>C/T<br>G/T<br>(WT[a])	N/A[b]	Substance specific function
CYP1A2	rs2069514 g.75038220 G>A in *1C,*1L<br>rs762551 g.75041917 C>A in *1F,*1L	G/A<br>A/A	*1F/*1L	Substance specific function
CYP2C8	rs10509681 c.1196A>G in *3<br>rs11572080 c.416G>A in *3<br>(rs1934951)	A/G<br>G/A<br>(WT[a])	*1A/*3	Substance specific function
CYP2C9	rs1799853 c. 430 C>T in *2<br>(rs1057910, rs9332131, rs7900194, rs28371685)	C/T<br>(WT[a])	*1/*2	Intermediate metabolizer (IM[c])
CYP2C19	rs4244285, rs4986893, rs12248560, rs28399504	WT[a]	*1/*1	Normal metabolizer (NM[d])
CYP3A4	rs2740574, rs2242480	WT[a]	*1A/*1A	Substance specific function
CYP3A5	rs776746	WT[a]	*3/*3	Poor metabolizer (PM[e])
HTR2A	rs6311 g.47471478C>T<br>rs6313 c. 102 C>T<br>rs7997012 c.614-2211T>C<br>(rs9316233, rs6314)	C/T<br>C/T<br>C/C<br>(WT[a])	N/A[b]	Substance specific function
NAT-2	rs1801280 c.341 T>C in *5,*5E,*5S<br>(rs1799930, rs1799931)	T/C<br>(WT[a])	*4/*5	Intermediate acetylator (IA[f])
OPRM1	rs1799971	WT[a]	N/A[b]	Substance specific function

[a]WT: Wild type, [b]N/A: Not applicable, [c]IM: Intermediate metabolizer, [d]NM: Normal metabolizer, [e]PM: Poor metabolizer, [f]IA: Intermediate acetylator

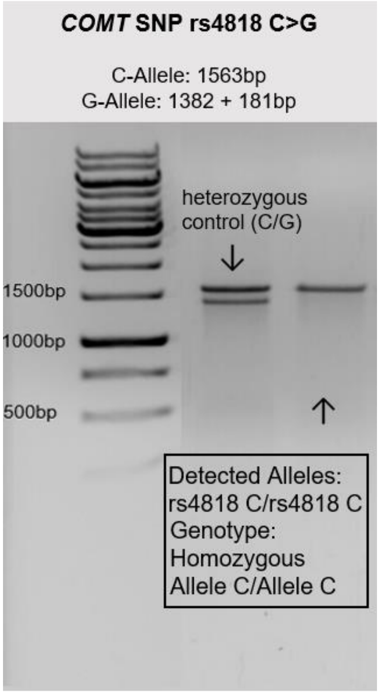

Supplementary Figure 1. Restriction fragment length polymorphism (RFLP) analysis results for *COMT* single nucleotide polymorphism (SNP) rs4818.
<u>Note:</u> Further detected protein bands of other patients were removed in this illustration.

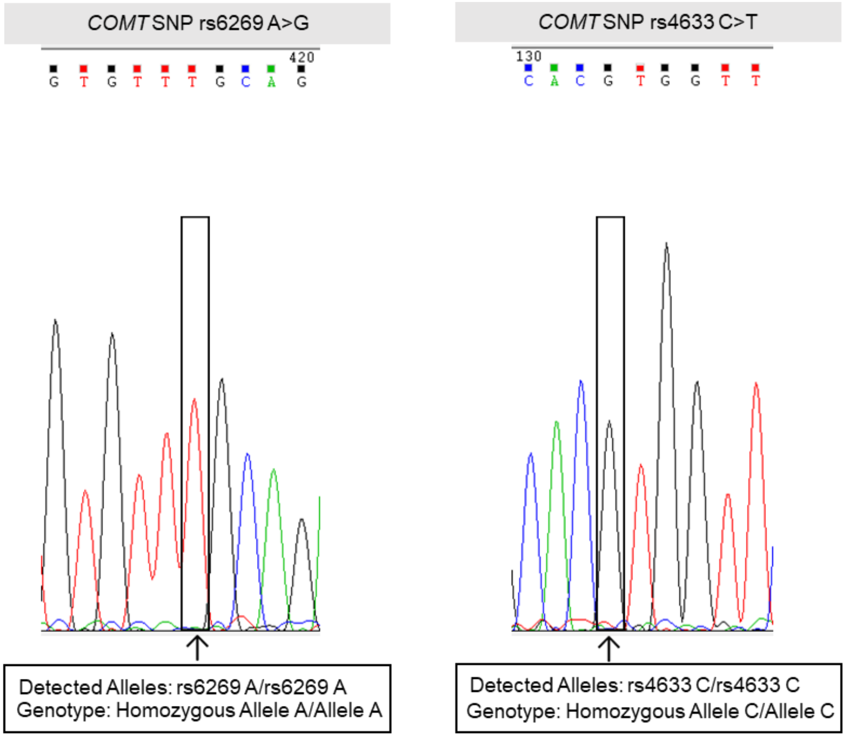

Supplementary Figure 2. Sanger sequencing results for *COMT* single nucleotide polymorphism (SNP) rs6269 and rs4633.
